# Digital Twin for a Multifunctional Technology of Flexible Assembly on a Mechatronics Line with Integrated Robotic Systems and Mobile Visual Sensor—Challenges towards Industry 5.0 †

**DOI:** 10.3390/s22218153

**Published:** 2022-10-25

**Authors:** Eugenia Mincă, Adrian Filipescu, Daniela Cernega, Răzvan Șolea, Adriana Filipescu, Dan Ionescu, Georgian Simion

**Affiliations:** 1 Department of Automation, Computer Science and Electrical Engineering, “Valahia” University of Târgoviște, 130024 Târgoviște, Romania; 2 School of Fundamental Sciences and Engineering, “Dunărea de Jos” University of Galați, 800008 Galați, Romania; 3 Department of Automation and Electrical Engineering, “Dunărea de Jos” University of Galați, 800008 Galați, Romania

**Keywords:** digital twin, real and virtual world, ML, MVSS, WMR, RM, multifunctional technology, flexible assembly, Industry 4.0 and 5.0

## Abstract

A digital twin for a multifunctional technology for flexible manufacturing on an assembly, disassembly, and repair mechatronics line (A/D/RML), assisted by a complex autonomous system (CAS), is presented in the paper. The hardware architecture consists of the A/D/RML and a six-workstation (WS) mechatronics line (ML) connected to a flexible cell (FC) and equipped with a six-degree of freedom (DOF) industrial robotic manipulator (IRM). The CAS has in its structure two driving wheels and one free wheel (2DW/1FW)-wheeled mobile robot (WMR) equipped with a 7-DOF robotic manipulator (RM). On the end effector of the RM, a mobile visual servoing system (eye-in-hand MVSS) is mounted. The multifunctionality is provided by the three actions, assembly, disassembly, and repair, while the flexibility is due to the assembly of different products. After disassembly or repair, CAS picks up the disassembled components and transports them to the appropriate storage depots for reuse. Disassembling or repairing starts after assembling, and the final assembled product fails the quality test. The virtual world that serves as the digital counterpart consists of tasks assignment, planning and synchronization of A/D/RML with integrated robotic systems, IRM, and CAS. Additionally, the virtual world includes hybrid modeling with synchronized hybrid Petri nets (SHPN), simulation of the SHPN models, modeling of the MVSS, and simulation of the trajectory-tracking sliding-mode control (TTSMC) of the CAS. The real world, as counterpart of the digital twin, consists of communication, synchronization, and control of A/D/RML and CAS. In addition, the real world includes control of the MVSS, the inverse kinematic control (IKC) of the RM and graphic user interface (GUI) for monitoring and real-time control of the whole system. The “Digital twin” approach has been designed to meet all the requirements and attributes of Industry 4.0 and beyond towards Industry 5.0, the target being a closer collaboration between the human operator and the production line.

## 1. Introduction

The main contribution of this article is the “digital twin” approach of an assembly technology that works on a mechatronic laboratory system, a technology that integrates and is assisted by robotic systems and a mobile visual servoing. The characteristics of the technology consist of the assembly of two different products, with the possibility of recovery by disassembling the components for the products that do not meet the quality (do not pass a quality test) or repair the products that allow this (partially satisfy the quality test) [1,2,3]. Digital twin targets two worlds, one virtual and another real. The virtual world consists of digital tools for modeling and simulating technology, specific actions, and operations. The real world is a transposition on the available hardware of all models and representations in the virtual world with remote monitoring [4,5]. 

In recent years, the industry has undergone a series of profound transformations that have led to unprecedented technological progress and a global evolution involving complex robotic systems; flexible, multifunctional manufacturing lines served by precision workstations; and efficient transportation systems and manipulation [6,7,8]. Studies and research results are based on increasing the diversification of manufacturing operations (assembly, processing, welding, etc.) on the same workstations and increasing productivity. All these have major implications on the quality of the final product, a quality that directly depends on the accuracy and precision of the manufacturing line [9,10,11].

The flexibility and optimization of manufacturing technologies have attracted the attention of researchers in the field. An important role was played by complex robotic systems, equipped with complex navigation systems and especially with visual sensors, which serve manufacturing technologies to increase productivity, recover, reuse, and revalue the components of a final product that does not correspond qualitatively [12,13,14,15,16]. Thus, the need arose for collaborative robotic systems that work by combining two service functions: the movement function performed by mobile robotics (WMRs) and the function of handling disassembled components (robotic manipulators) [14,17].

The latest technology digital twin is now attracting attention in various fields, “twin” of “digital”. In other words, it is a technology that digitally reproduces the real world. It is said to be the key to the industry of the future, especially the future of making that is a digital twin for manufacturing. A digital twin is a virtual representation of a real-world product or asset, with the benefit of continuous, real-time data from a product or a manufacturing technology. Digital twins provide insights to increase productivity, to improve product quality, components and subassemblies recovery, reduce downtime, optimization, and control of manufacturing processes [4,5,18].

In a digital twin, there are several solutions for digital transformations in manufacturing technologies [19]:

- Additive manufacturing: Additive manufacturing, also known as 3D printing, is a process used to create a physical or 3D object by layering materials one by one based on a digital model [20];
- Autodesk software: Advanced manufacturing software allows you to make anything you want;
- Augmented reality: Augmented reality, virtual reality, and mixed reality involve immersive technologies to revolutionize data interaction and project collaboration between team members and how people interact with their data;
- Digital transformation: Digital transformation means convergence for connecting organizations using data, while bridging the gap to bring together different disciplines, such as computer aided design (CAD), computer aided manufacturing (CAM), or computer aided engineering (CAE), collaboration by accessing data via the cloud, for connecting your entire manufacturing ecosystem and automation for removing the delays, and using generative design and robotics to streamline multiple processes and accelerate product development;
- Generative design: Generative design quickly generates high-performing design alternatives and multiple solutions to solve the needs;
- Robotics: Robot programming software for manufacturing has a great impact on the collaboration between humans and robots [21,22];
- Simulation: Simulation software allows predicting, validating, and optimizing products using accurate analysis.

In our digital twin approach, the virtual world consists of tasks assignment, planning, and synchronization of A/D/RML with integrated robotic systems, IRM, and CAS, the last one having as components: PeopleBot WMR, Cyton 1500 RM, and MVSS. CAS can also be assimilated to a mobile cyber-physical system (MCPS), where the intelligent system is a computer system in which a mechanism is controlled and monitored by computer-based algorithms. In cyber-physical systems, physical and software components are deeply intertwined, capable of operating at different spatial and temporal scales, exhibiting multiple and distinct behavioral modalities, and interacting with each other in context-changing ways. Additionally, the virtual world includes hybrid modeling and simulation with synchronized hybrid Petri nets (SHPN), modeling and implementation of the MVSS, simulation in MobileSim of the TTSMC of the CAS, IKC of the RM, and the Graphic User Interface (GUI} for monitoring of real-time control, so that the whole system becomes fully automated.

The real world consists of communication, synchronization, monitoring, and control for a multifunctional technology for flexible manufacturing that works on a laboratory system and integrates several subsystems, namely an assembly/disassembly mechatronics line (A/DML) and an assembly/disassembly flexible cell (A/DFC) with an integrated 6-DOF IRM. A/DML and A/DFC will be referred to as A/D/RML assisted by a CAS that consists of an autonomous robotic system, which is a WMR equipped with a 7-DOF RM and an *eye-in-hand* MVSS located on the end effector. All these subsystems are equipped with PLCs, wired and wireless communication devices, infrared, inductive, and optical sensors, and electric and pneumatic actuators. The technology allows the assembly of two different products and complete disassembly or repair of the product that fails quality tests. Components resulting from disassembly or repair are recovered by CAS and deposited for reuse.

Thus, the paper claims several concepts specific to Industry 4.0, such as: “Digital twin” of real-world application, MCPSs, IoT, cloud storage to efficiently increase autonomy, big data collection and manipulation, smart manufacturing, efficient production lines and smart products, communication security, and cybersecurity [23,24].

Also in this work, a series of challenges are launched towards Industry 5.0 in the sense of overcoming the problems associated with removing human workers from various processes. At the same time, Industry 5.0 aims to pair humans and workstations to further utilize human brain power and creativity to increase process efficiency by combining workflows with intelligent systems. While the main concern in Industry 4.0 is about automation, Industry 5.0 will be a synergy between humans and autonomous machines. These challenges, through the “Digital twin” approach of the multifunctional technology of flexible manufacturing, would be the following: networked sensor data interoperability, virtual training, intelligent autonomous systems, including MCPSs, advances in detection technologies, and knowledge of workstations [25,26,27].

The rest of the paper is organized as follows: the hardware of the multifunctional flexible manufacturing technology, consisting of the hardware architecture of A/D/RML assisted by CAS, is laid out in Section 2 with: hardware architecture, assumptions, flexible manufacturing, and multifunctional technology. The virtual world as a digital counterpart of multifunctional flexible manufacturing technology is presented in Section 3 with a digital counterpart regarding assembly, disassembly, repair, and CAS. Real counterpart control of multifunctional technology running on A/D/RML assisted by CAS is presented in Section 4 with communication and control of A/D/RML, synchronization and control of CAS, control of MVSS, control of Cyton RM, and control of CAS PeopleBot WMR assisting A/D/RML during disassembly. Some remarks and discussions about the digital twin approach of the multifunctional flexible manufacturing technology can be found in Section 5, Discussion. In the final section, Conclusions, the goals pursued by the approach and research are presented.

## 2. The Hardware Architecture of Multifunctional Flexible Manufacturing Technology Running on A/D/RML Assisted by CAS

### 2.1. Hardware Architecture

The real world of the multifunctional technology of flexible manufacturing working on A/D/RML assisted by CAS consists of three main components and subsystems, which are synchronized to work together and act as a flexible manufacturing line that performs several operations, such as the assembly of two different products (workpieces) with disassembly, repair, and recover functionality [2,3].

**Figure 1:**
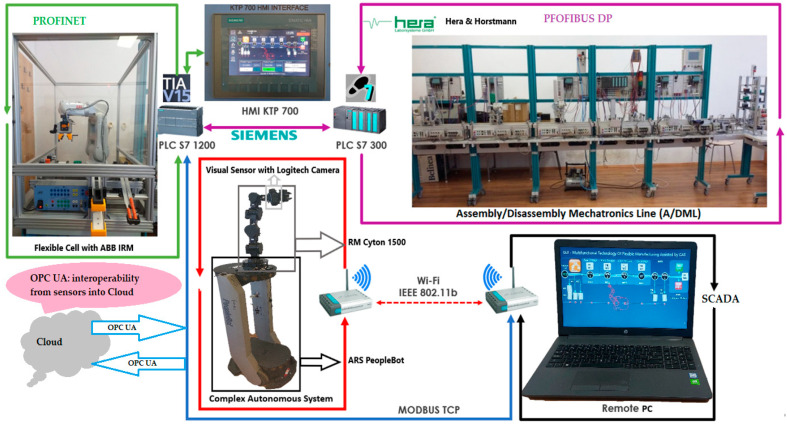
Control structure of A/DML Hera & Horstmann, FC with ABB IRM and CAS with PeopleBot WMR, and Cyton 1500 RM.

**Figure 2:**
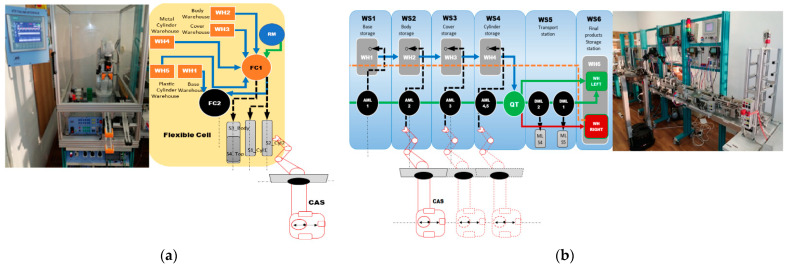
A/D/RML assisted by CAS: (**a**) Flexible cell station with 6-DOF ABB IRB120; (**b**) A/DML Hera & Horstmann.

**Figure 3:**
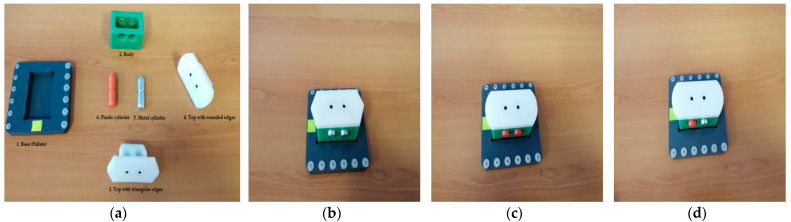
(**a**) workpiece parts, assembled workpieces: (**b**) workpiece with metal cylinders; (**c**) workpiece with plastic cylinders; (**d**) workpiece with different material cylinders.

The structure of the A/D/RML is presented in [2] and is shown in Figure 1. The major components are:

- FC with 6-DOF ABB IRB120 station used for assembly, disassembly, and repair of the workpieces with buffer, handling, processing, and transport capability, Figure 1 and Figure 2a. FC station with 6-DOF ABB RM used for assembly, disassembly, and repair of the workpieces with buffer, handling, processing, and transport capability. FC has as its main components a 6-DOF RM pick-and-place, a Siemens S7-1200 PLC, and a controlled assembly/disassembly unit, which handles the supply of workparts (components) for the workpiece product type 1 (WP1), disassembly, and repair for the second workpiece (WP2);
- A/DML is a 6-workstation (6-WS) Hera & Horstmann ML, which together can perform the following operations: A/D, transport, checking, and storage of assembled workpieces, Figure 1 and Figure 2b. It is a laboratory mechatronic line for didactic and research use. The parts to be assembled are: (1) pallet (base), (2) body, (3) top with triangular edges, (4) top with rounded edges, (5) metal cylinder and (6) plastic cylinder, Figure 3a. The assembled workpieces with metal, plastic, or different material cylinders are shown in Figure 3b–d, respectively. The stations are equipped with inductive sensors that work as position or verification sensors. WS1 is the station where the warehouse with the pallets is located. Base is the support of the workpiece and is equipped with a 6-bit coding system, which offers many large codes, identified by means of inductive sensors. The workpiece that is assembled on the A/DML consists of four parts, (1), (2), (4), (5), and (6), being subjected to assembly, testing, sorting, and storage. The disassembly is not used in this paper. WS2 is the station where the warehouse with component (2), WH2, is located. WS3 is the station where the warehouse with component (4), WH3, is located. Component (3) is assembled only in FC. In WS4, the warehouse with components (5) and (6), WH4, and the test post for the cylinders, QT, are located. WS5 is a multidirectional conveyor station. WS 6 is the final two racks storage station. The stations are equipped with conveyor belts and inductive position sensors, which, by means of pneumatic actuators, perform localization of the workpiece. The control architecture is a distributed one consisting of: SIEMENS S7-300 PLC with a CP 314C-2 DP series processor, CP 343-2 communication module, and ET 200S IM 151-1 distributed on each station, having digital and analog I/O for signals from sensors and commands to actuators. All are connected to PROFIBUS DP. HMI TP 177 is connected to the PROFIBUS DP as terminal, used to select commands and view assembled final products, and stored in WS6.
- CAS is PeopleBot WMR equipped with a 7-DOF Cyton 1500 RM used for recovery and transport/return operation of the disassembled workparts and a mobile VSS [2]. The CAS, shown in Figure 1, is composed of the following elements: a 7-DOF Cyton 1500 RM equipped with an *eye-in-hand* type of MVSS, using a high-definition camera (visual sensor), both being connected to a computer via USB, and synchronously communicating with the A/D/RML over Wi-Fi. The RM is placed on the PeopleBot, which is a WMR with two driving wheels and one free wheel (2DW/1FW). The CAS is used to transport the recoverable workpart, picked up by the Cyton 1500 RM, to the appropriate storage warehouse if the assembled piece WP2 has failed the quality test and will be disassembled or repaired.

### 2.2. Assuptions Regarding Hardware Arhitecture

The technology on A/D/RML assisted by CAS and *eye-in-hand* VSS, described above, depends on aspects such as operation modes, operation lengths, and types of finished products (Figure 2) [2]. Therefore, for FC, A/DML, CAS, and VSS, some assumptions must be established for controlling the whole system.

**Assumption** **H1.** *The A/D/RML is a single-model line, by the nature of the product, paced line (transfers between the workstations are synchronous), by the operation mode, and deterministic line, by the nature of operation times (times known certainly).*

**Assumption** **H2.** *The number of the A/D/RML workstations involved in A/D/R is previously known and will remain unchanged (FC with ABB IRM and 6-WS A/DML, Hera & Horstmann).*

**Assumption** **H3.** *The workstations of the A/D/RML have a linear distribution, FC and WS1 to WS6.*

**Assumption** **H4.** *The left side (in green WH left) of the WS6 station is the warehouse where good products are stored, while the right side (in red WH right) is the warehouse where products that do not pass the quality test are stored, need to be disassembled, or repaired.*

**Assumption** **H5.** *One CAS assists the A/D/RML, having mounted an RM, used for picking up, transport, and depot of the workparts.*

**Assumption** **H6.** *One eye-in-hand MVSS camera is mounted on the RM.*

**Assumption** **H7.** *CAS displacement is without obstacles and with the same constant speed.*

### 2.3. Flexible Assembly

A/D/RML is a flexible manufacturing line because it assembles two different products, referred to as workpiece 1 (WP1) and workpiece 2 (WP2). WP1 is the workpiece with the cover part having triangular edges (Figure 3a,b) and is assembled in the FC with the ABB IRM, Figure 2a. WP2 is the workpiece with the top part having round edges (Figure 3a,c,d) and is assembled on the Hera & Horstmann ML, Figure 2b.

### 2.4. Multifunctional Manufacturing Technology, Assembly, Disassembly, and Repair

#### 2.4.1. Assembly

WP1 is assembled in the FC by the ABB IRM, taking from FC’s warehouses the components in order: base, body, top (cover) and cylinders, metal or plastic. First, the base is positioned on the conveyor belt, then the rest of the product is assembled in a separate location from the FC, and it is moved by the ABB IRM onto the base. Finally, WP1 is transferred along the Hera & Horstmann ML, and it is stored on the left side of the WS6 station. In Figure 2a is presented the FC equipped with the ABB IRM and the assembly process structure. The A/DML Hera & Horstmann realizes the transfer to the left side rack of WS6. 

WP2 is randomly assembled with the two cylinders, in WS1 to WS4 of the A/DML, as shown in Figure 3b. The components to be assembled are base (work part carrier), body, top (cover), metal cylinder, and plastic cylinder.

#### 2.4.2. Disassembly

**Figure 4:**
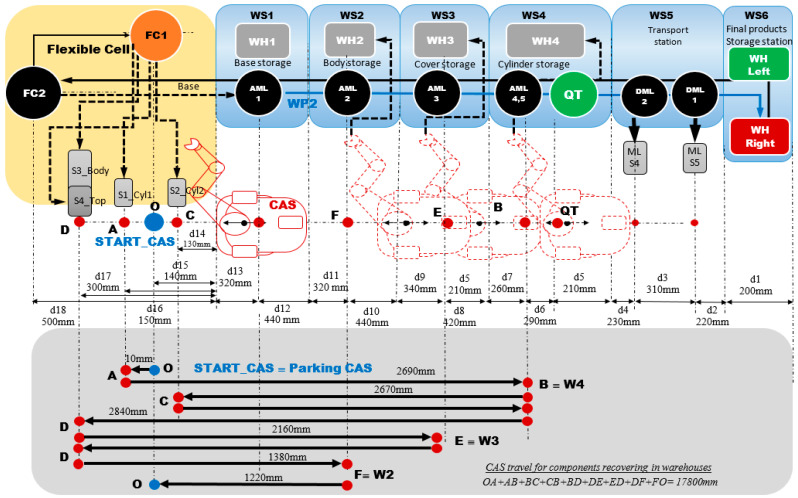
A/D/RML and distances traveled by CAS for part recovery corresponding to the disassembly function.

WP2, being considered scrap (it has two plastic cylinders, Figure 3b), is taken over by the elevator of the WS6 and positioned on the transport station WS5. It is transported along the A/DML Hera & Horstmann to the FC (FC2), Figure 4.

#### 2.4.3. Repair

**Figure 5:**
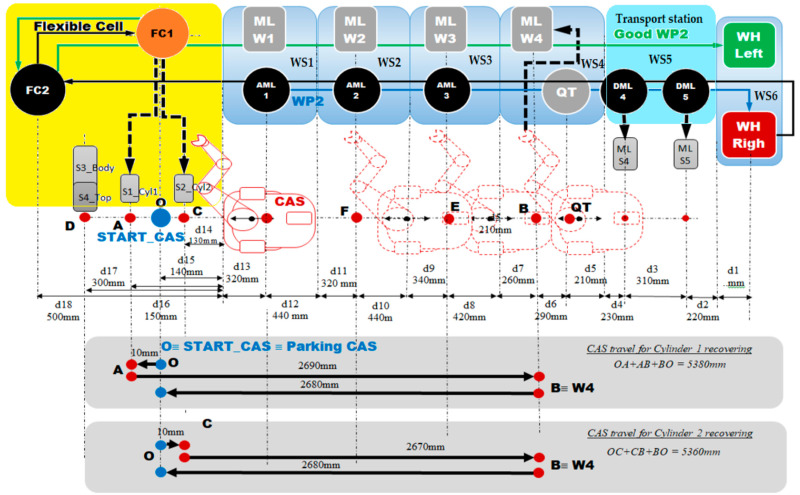
A/D/RML and distances traveled by CAS for cylinder recovery corresponding to the repair function.

WP2, having cylinders of different materials (Figure 3d), is taken over by the elevator of WP6 and positioned on WS5. It is transported along the Hera & Horstmann ML to the FC (FC2). The ABB IRM disassembles the plastic cylinder (on FC1), Figure 5.

## 3. The Virtual World as a Digital Counterpart of Multifunctional Flexible Manufacturing Technology

This chapter will cover the most important components of the virtual world, the digital duplication of multifunctional technology, running on A/D/RML assisted by CAS, and the mobile visual sensor: task assignment, planning, communication, synchronization, SHPN modeling and simulation, and CAS modeling and simulation. The need for the SHPN model is justified by the necessity of collaboration between the mechatronics line and the CAS that serves it. In more detail, in this approach, the hybrid Petri net (HPN), which is the SHPN without the synchronization signals from the sensors, is autonomously modeled, simulated, and tested. Compatibility between A/D/RML and CAS is necessary because both have physical characteristics and constraints that should be considered. 

The proposed SHPN models for assembly, disassembly, and repair are indispensable for simulation and represent the stage that precedes the implementation of real-time control [12,13,14].

**Figure 6:**
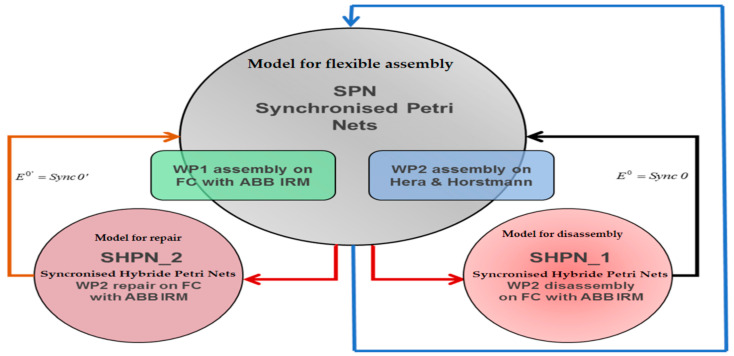
The block scheme of the interconnection through synchronization signals of the three operation models.

The simulation of SHPN models makes it possible to monitor the evolution of the integrated system, A/D/RML served by CAS, in the state space, because of the transitions between states, the evolution being consistent with the constructive elements. The inputs in SHPN, imposed in the modeling stage, are programming of operations on A/D/RML, their durations, CAS movement distances and durations, manipulation durations for each operation, precisely estimated positioning times of the Cyton 1500 RM, for picking up the component from the disassembly location, and transporting and storing it in the warehouse. Precise positioning times represent a major uncertainty in our approach due to existing constructive constraints that could compromise real-time control. The solution found for this problem is based on a mobile eye in hand visual servoing system. The SHPN models proposed for this multifunctional technology are used in simulation and thus provide decisional information to be used in the control structure. The control structure of the whole system is based on synchronization. The multifunctional system has both continuous and discrete dynamics for the three interconnected processes. Therefore, the model for the multifunctional process is the synchronized connection between the three models. The three models are interconnected through synchronization signals, as shown in Figure 6 [3,12].

To ensure the best performances of the real-time implementation of the control structure of the multifunctional system, the SPN and SHPN models are used. The simulation results of the SPN and SHPN models provide the possibility to monitor the evolution of the integrated system because of transition triggering. The simulation results analysis offers the possibility to improve the system performances.

### 3.1. Virtual Digital Counterpart Regarding Assembly

#### 3.1.1. Assumptions

**Assumption** **A1.** *Two types of workpieces are assembled: WP1 in FC with ABB IRM and WP2 in Hera & Horstmann ML. The assembly operations of WP1 are executed in FC. The assembly operations of WP2 are executed on Hera & Horstmann ML.*

**Assumption** **A2.** *All conditions and parameters of the assembly are initially known, including task durations.*

**Assumption** **A3.** *WP2 that fails the quality test is stored in the right side of WS6.*

**Assumption** **A4.** *By convention, it is assumed that the WP2 fails the quality test if it contains either plastic cylinders or different materials.*

#### 3.1.2. Task Assignment, Planning, and Synchronization

The assembly of WP1 is made by the ABB IRM, taking from CF warehouses the components in order (Figure 3a: base, body, top, and cylinders, metal or plastic). First, the base is positioned on the conveyor belt (on FC2), then the rest of the product is assembled in a separate location of the FC (on FC1), then it is moved by the ABB IRM onto the base (on FC2). Finally, WP1 moves along the Hera & Horstmann ML and is stored on the left side of the WS6 station. The graphical user interface (GUI), on the HMI pen, allows selection for assembly between plastic cylinders and metal cylinders. Due to this fact, the WP1 product is of good quality and, for this reason, is stored in the rack on the left side of the WS6 station. The WP2 product is randomly assembled with the two cylinders and is subjected to the quality test on the WS4 station. To evaluate the quality for the WP2 product, the convention is that a WP2 product assembled with both metal cylinders is considered of good quality and is stored on the left side of the WS6 station.

The WP2 product that contains both plastic cylinders (Figure 3c) is considered scrap product, and it is stored in the rack on the right of the WS6 station. This WP2 will be disassembled for component recovery (Figure 4). The WP2 product having different material cylinders (Figure 3d) is also deposited in the rack on the right, and it will be repaired by replacing the plastic cylinder with a metal one (Figure 5). 

**Figure 7:**
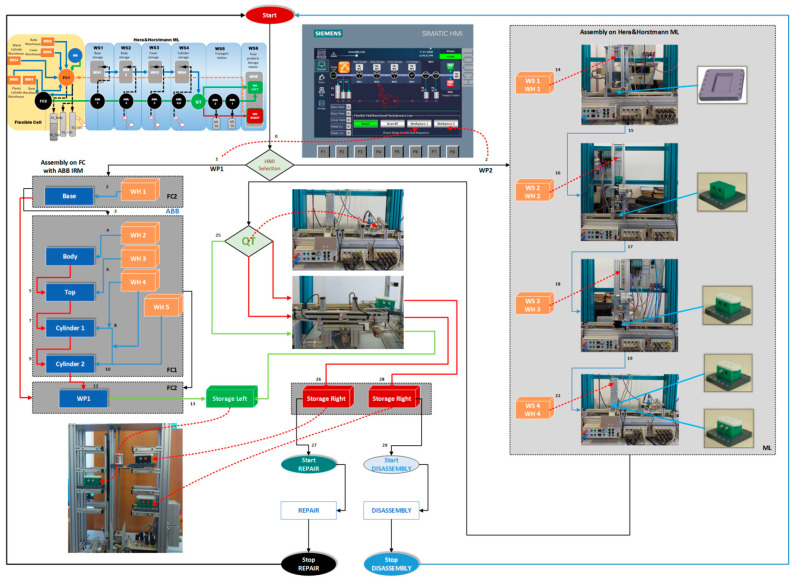
Task planning and synchronization for assembly: WP1 on FC and WP2 on A/DML.

In Figure 7 is presented the block diagrams with the planning and synchronization of tasks for assembly [28,29,30,31].

#### 3.1.3. SPN Model, Formalism, and Simulation

**Figure 8:**
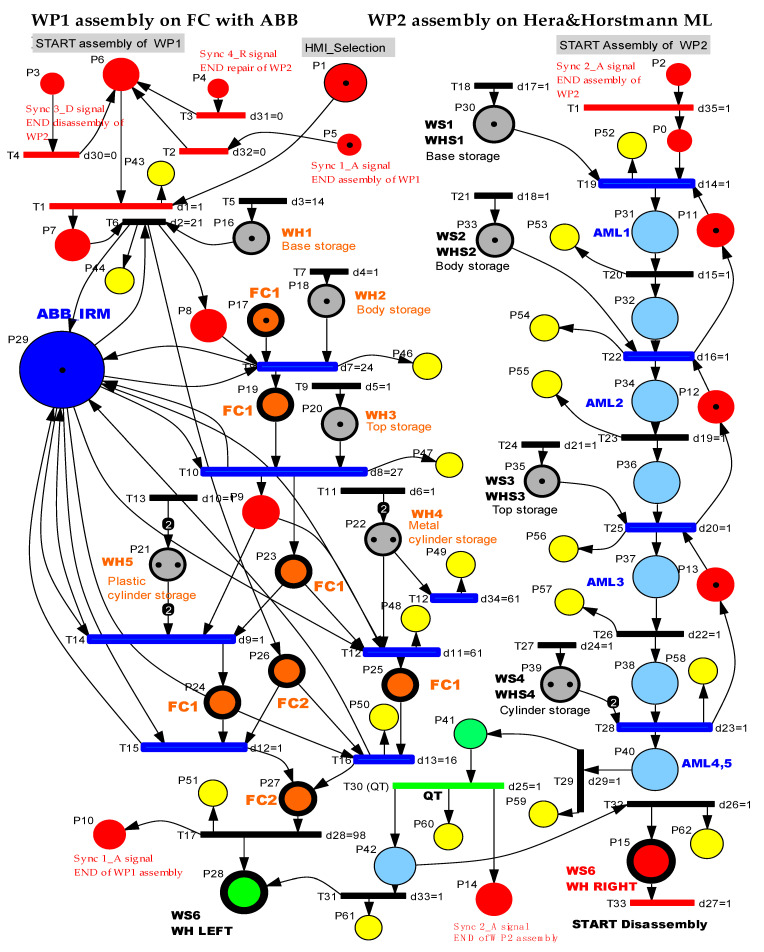
SPN, synchronized Petri net, for WP1 assembly on FC with ABB IRM and WP2 assembly on A/DML Hera & Horstmann.

The use of the SPN model for the assembly process for the WP1 on the FC and WP2 on the ML is justified by the necessity of synchronization between the two resources: Hera & Horstmann ML and the flexible cell FC [2,3,12,13,14]. The need for synchronization is determined by the fact that the FC is also implied in the disassembly process and in the repair process, and the storage and the quality control of the workpiece is performed by the Hera & Horstmann ML. In this approach, the synchronized Petri net (SPN), is obtained using synchronized signals from sensors and is modeled, simulated, and tested in autonomous mode. The SPN model, presented in Figure 8, corresponds to the two parallel assembly processes, and the synchronization is analyzed because both resources must meet the constraints that enable them to accomplish the desired tasks.

The SPN is defined by:

(1) $$\mathrm{SPN}=\langle \begin{array}{ccc} \mathrm{TPN}, & E^{0}\cup E^{0^{\prime }}, & \mathrm{Sync} \end{array}\rangle ,$$

where TPN is the timed Petri net, defined as follows:

(2) $$\mathrm{TPN}=\langle P,T,\mathrm{Pre},\;\mathrm{Post},m_{0},\mathrm{tempo}\rangle .$$

The elements of the TPN from (2) are:

- P is the places set partitioned in:

(3) $$P=\left\{P_{\mathrm{ctr}},P_{a},P_{\mathrm{QT}},P_{monitoring}\right\},$$

where:

(4) $$P_{\mathrm{ctr}}={\left\{P_{i}\right\}}_{i=\bar{1,13}}$$

represents the state set associated to the control functions of the decision actions,

(5) $$P_{a}={\left\{P_{j}\right\}}_{j=\bar{14,40}}$$

represents the set of the discrete places modeling the flexible assembly operations for the two work pieces (WP1 and WP2),

(6) $$P_{\mathrm{QT}}=\left\{P_{41},P_{42}\right\}$$

represents the states set associated to the quality testing (QT) operations (in WS4) of the workpiece,

(7) $$P_{\mathrm{monitoring}}=\left\{P_{43},...{,P}_{51}\right\}\cup \left\{P_{52},..{,P}_{62}\right\}$$

represents the states set associated to the monitoring of the successive assembly actions for WP1 and WP2.

- T is the transitions set partitioned in:

(8) $$T=\left\{T_{a},T_{QT},T_{storage}\right\},$$

where:

(9) $$T_{a}={\left\{T_{i}\right\}}_{i=\bar{1,35}}$$

is the set of the discrete transitions for the two workpiece (WP1, WP2) assembly,

(10) $$T_{QT}=\left\{T_{29},T_{30}\right\}$$

is the set of the discrete transitions associated to the QT functions,

(11) $$T_{storage}=\left\{T_{31},T_{32}\right\}$$

is the set of the discrete transitions associated to the storage functions in the two warehouses WH Right and WH Left.

- For WP1 assembly on FC with ABB IRM, the monitoring places in the set (7) monitor the transitions in the set (8) as follows: P43(T1_monitoring), P44(T6_monitoring), P46(T8_monitoring), P47(T10_monitoring), P48(T12_monitoring), P49(T12_monitoring), P50(T16_monitoring), P51(T17_monitoring).
- For WP2 assembly on Hera & Horstmann ML, the monitoring places in the set (7) monitor the transitions in the set (8) as follows: P52(T19_monitoring), P53(T20_monitoring), P54(T22_monitoring), P55(T23_monitoring), P56(T25_monitoring), P57(T26_monitoring), P58(T28_monitoring), P59(T29_monitoring), P60(T30_monitoring), P61(T31_monitoring), P62(T32_monitoring).
- $Pre:P\times T\to Q_{+}$ is the input incidence function.
- $Post:P\times T\to Q_{+}$ is the output incidence function.
- $m_{0}$ is the initial marking of the SPN corresponding to the initial state of the modeled process.
- $tempo:T\to Q_{+}\cup \left\{0\right\}$ is a function that defines the timings associated to the transitions.

(12) $$E=E^{0}\cup E^{0^{\prime }}$$

is the set of external events:

(13) $$E=\left\{Ed^{1},Ed^{2},\;Ed^{3},Ed^{4}\right\}\cup \left\{e\right\}.$$

The Sync application in definition (1) is a function from the set of discrete disassembly transitions to the set of external events joined with the neutral element e

(14) $$Sync\;:\left\{T_{2},\;T_{3},\;T_{4},\;T_{19}\right\}\to \left\{Ed^{1},\;Ed^{2},\;Ed^{3},\;Ed^{4}\right\}\cup \left\{e\right\},$$

(15) $$Sync\;1\_A:T_{2}\to \left\{Ed^{1}\right\},$$

(16) $$Sync\;2\_A:T_{19}\to \left\{Ed^{2}\right\},$$

(17) $$Sync\;3\_D:T_{4}\to \left\{Ed^{3}\right\},$$

(18) $$Sync\;4\_R:T_{3}\to \left\{Ed^{4}\right\}.$$

- Ed^1^ = Svnc1_A is synchronization signal for: (START assembly of WP1) with (END assembly of WP1).
- Ed^2^ = Svnc2_A is synchronization signal for: (START assembly of WP2) with (END assembly of WP2).
- Ed^3^ = Svnc3_D is synchronization signal for: (START assembly of WP1) with (END disassembly of WP2).
- Ed^4^ = Svnc4_R is synchronization signal for: (START assembly of WP1) with (END repair of WP2).

**Figure 9:**
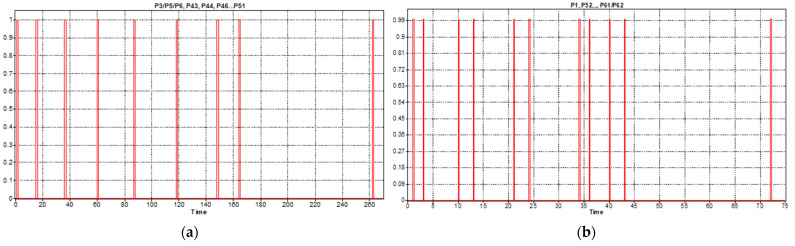
The monitoring signals of the successive actions for: (**a**) WP1 assembly; (**b**) WP2 assembly.

The results of the SPN model simulation in Sirphyco [32] for the assembly processes of the WP1 and WP2 simulation are presented in Figure 9a,b, respectively.

The monitoring signals are used to synthesize the synchronization signals at the control level of the multifunctional system.

### 3.2. Virtual Digital Counterpart Regarding Dissasembly

#### 3.2.1. Assumptions

**Assumption** **D1.** *All conditions and parameters of the disassembly function are initially known, including task durations.*

**Assumption** **D2.** *The convention assumes that WP2 does not pass the quality test, being liable to disassembly if it contains both plastic cylinders.*

**Assumption** **D3.** *WP2 that fails the quality test is stored in the right side of WS6, and its disassembly processes starts immediately after.*

**Assumption** **D4.** *The disassembly operations of WP2 are executed on FC.*

#### 3.2.2. Task Assignment, Planning, and Synchronization

The ABB IRM disassembles it in the established order: cylinder 1 (left), cylinder two (right), top, and body (on FC1), letting them slide on the corresponding trough. The base is transported back to WH1 located on ML, where the piston pushes it into the storage warehouse. 

CAS takes over each component in order, cylinder 1, cylinder 2, body, and top, transporting it to the appropriate storage warehouse on the Hera & Horstmann ML (Figure 4). The precision positioning of the CAS is performed with the eye-in-hand VSS (Figure 1). 

**Figure 10:**
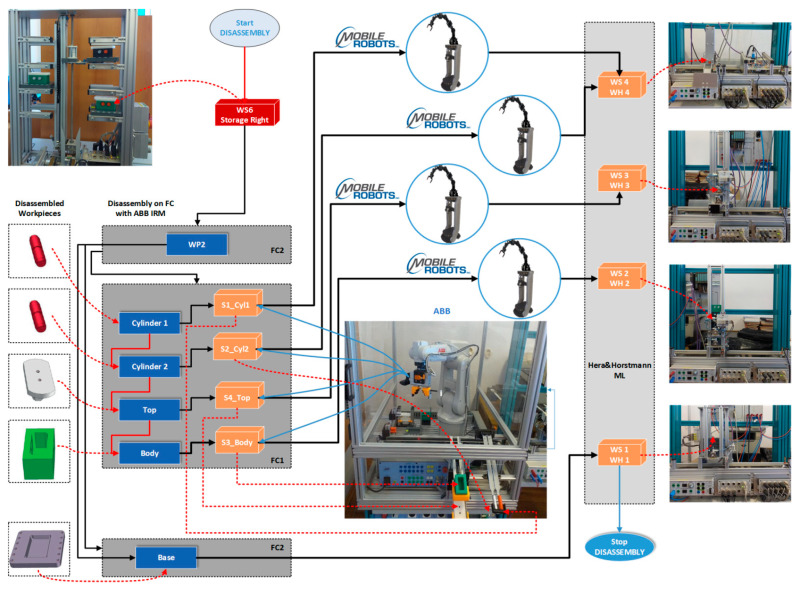
Task planning and synchronization for disassembly of the WP2 on FC with ABB IRM.

In Figure 10 is presented the block diagrams with the planning and synchronization of tasks for disassembly [28,29,30,31]. The disassembly function is associated with the recovery and reuse of components.

#### 3.2.3. SHPN Model, Formalism, and Simulation

**Figure 11:**
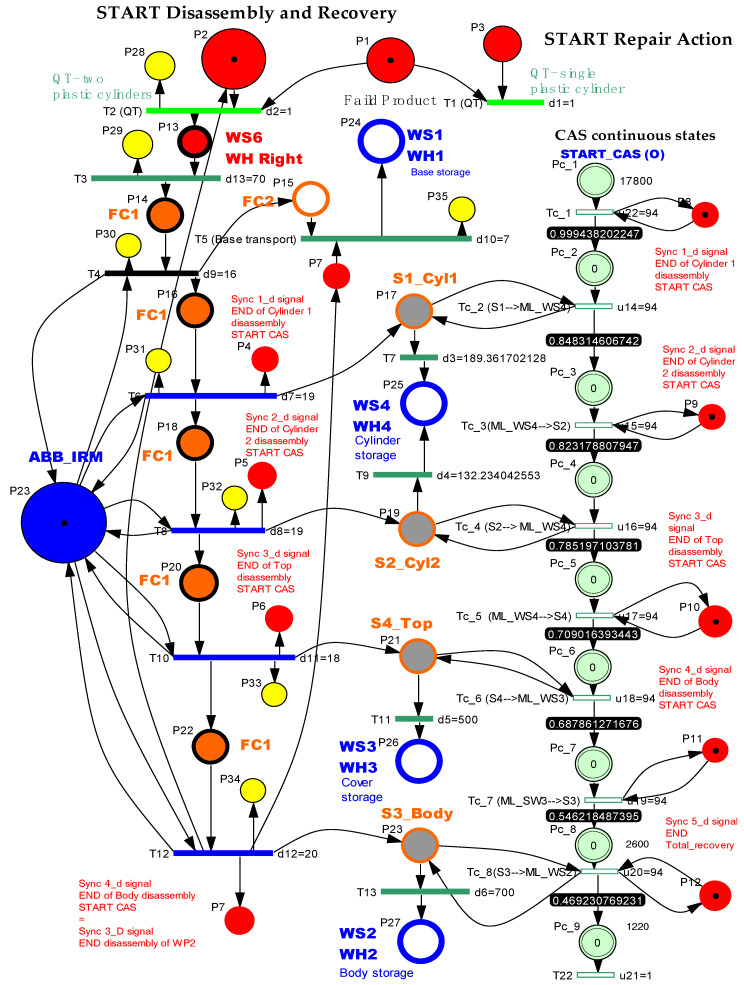
The SHPN_1 model for the disassembly of the WP2 on FC with ABB IRM.

The SHPN_1 model from Figure 11, based on task planning from Figure 10, is an oriented graph described with the synchronized hybrid Petri nets (SHPN) formalism. The SHPN_1 model describes both discrete and continuous dynamics corresponding to the disassembly functionality. The discrete model corresponds to the disassembly operations on FC with ABB IRM, while the continuous one corresponds to the CAS displacements for recovery and storage of the components. Thus, the model becomes a hybrid one [2,3,12,13,14].

The SHPN_1 model for the disassembly process is a triplet

(19) $$SHPN\_1=\langle \begin{array}{ccc} HPN, & E^{0}, & Sync \end{array}\rangle ,$$

where: HPN is the hybrid Petri net model, $E^{0}$ is a set of external events, and Sync is an application from the set of transitions to that of external events. 

The HPN is a septuplet:

(20) $$HPN=\langle T,P,\mathrm{Pr}e,Post,m_{0},h,tempo\rangle ,$$

where:

- T is the transitions set partitioned in:

(21) $$T=\left\{T_{d},T_{c}\right\},$$

with

(22) $$T_{d}=\left\{T_{disassembly},T_{QT},T_{storage}\right\},$$

where

(23) $$T_{disassembly}={\left\{T_{i}\right\}}_{i=\bar{3,13}}\backslash \left\{T_{7},T_{9},T_{11},T_{13}\right\}$$

is the set of the discrete transitions for the WP2 disassembly tasks,

(24) $$T_{QT}=\left\{T_{1},T_{2}\right\}$$

is the set of the discrete transitions for the quality test (QT) tasks,

(25) $$T_{storage}=\left\{T_{7},T_{9},T_{11},T_{13}\right\}$$

is the set of the discrete transitions associated to the storage tasks in the two warehouses, WH Right and WH Left,

(26) $$T_{c}=\left\{T_{c\_1},..,T_{c\_8}\right\}$$

is the set of continuous transitions of the mobile robot (CAS) used to model the pick-up/transport/recovery in the corresponding station’s warehouse of the disassembled components;

- P is the places set partitioned in:

(27) $$P=\left\{P_{d},P_{c}\right\},$$

with

(28) $$P_{d}=\left\{P_{ctr},P_{disassembly},P_{monitoring}\right\}={\left\{P_{i}\right\}}_{i=\bar{1,36}},$$

where

(29) $$P_{\mathrm{ctr}}={\left\{P_{i}\right\}}_{i=\bar{1,12}}$$

represents the set of places associated with the control functions related to some decision-making actions,

(30) $$P_{\mathrm{disassembly}}={\left\{P_{j}\right\}}_{j=\bar{13,27}}$$

represents the discrete places set modeling the disassembly operations on the FC,

(31) $$P_{\mathrm{monitoring}}=\left\{P_{28},\ldots ,P_{35}\right\}$$

represents the set of places associated with the monitoring function of the successive disassembly actions,

(32) $$P_{c}=\left\{P_{c\_1},..,P_{c\_9}\right\}$$

represents the set of continuous places of the mobile robot in pick-up/transport/components recovery actions in warehouses.

Each place in the set (31) monitors a certain transition in the set (23) as follows: P28(T2_monitoring), P29(T3_monitoring), P30(T4_moitoring), P31(T6_monitoring), P32(T8_monitoring), P33(T10_monitoring), P34(T12_monitoring), P35(T5_minitoring).

- Pre: P × T → Q_+_ is the input incidence function.
- Pre: P × T → Q_+_ is the input incidence function.
- Post: P × T → Q_+_ is the output incidence function.
- $$tempo:T\to Q_{+}\cup \left\{0\right\}$$
  is function that defines the timings associated to the transitions.

The SHPN model, related to the total disassembly, describes using the timed Petri net (TPN) model, the actions of disassembly/transport on the conveyor and handling of WP2. The TPN is added to the continuous places modeling of the CAS position variation during the transfer operations $P_{c\_1},..,P_{c\_9}$. The hybrid appearance results from adding the continuous places to the timed places. 

The displacement sequences of CAS are synchronized with the transitions of the disassembly tasks $T_{6},T_{8},T_{10},T_{12}$ as follows:

(33) $$E^{0}⊇\left\{Sync1\_d,Sync2\_d,Sync3\_d,Sync4\_d\right\},$$

(34) $$E^{0}={\left\{E^{i}\right\}}_{i=\bar{1,5}}\cup \left\{e\right\},$$

(35) $${\left\{E^{i}\right\}|}_{i=\bar{1,5}}={\left\{Sync\;i\right\}|}_{i=\bar{1,5}},$$

where {e} is the neutral event considered to synchronize the transitions set $T\backslash \left\{T_{6},T_{8},T_{10},T_{12}\right\}$ whose firing is not externally conditioned.

The end of the process of total disassembly is synchronized with the start of a new assembly process through the signal:

(36) $$Ed^{3}=Sync\;3\_D,$$

which is the synchronization signal for: (START assembly of WP1) with (END disassembly of WP2).

**Figure 12:**
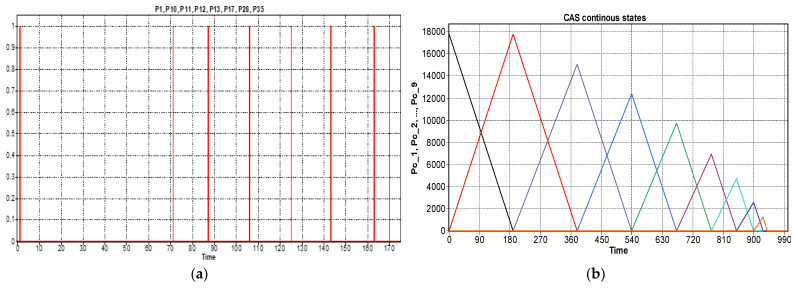
(**a**) The monitoring signals for the successive actions of the WP2 disassembly. (**b**) The continuous places evolution of the CAS (PeopleBot WMR) places: P_c_1_,..,P_c_9_.

The simulation results, obtained in Sirphyco, for the SHPN_1, are presented in Figure 12a,b. It was considered that the position of CAS has a continuous variation over time, during the disassembly and recovery of the components.

The markings $P_{c\_1},..,P_{c\_9}$ represent the distance remaining to be covered by the CAS starting from the parking position, until the completion of a complete cycle of disassembly-recovery of the components in the warehouses of the line: WS2, WS3, and WS4. For a complete disassembly–recovery cycle of the components, the CAS moves at a constant speed successively performing the following sequences (Figure 4): moving from the parking position (O) with positioning to S1 (S1≡(A)) for picking up the first cylinder and then CAS moves to the WH4 (WH4≡(B)) warehouse to deposit it. Afterwards, CAS returns to position S2 (S2≡(C)) to take over the second cylinder and then moves back to WS4 to deposit it. Then follows the CAS shift to the S4 (S4≡(D)) position to take over the cover (op) and deposit it in the WH3(WH3≡(E)). Finally, the CAS shifts to the S3 (S3≡(D)) body pick-up and deposits it in WH2 (WH2≡(F)) storage. Pallet pick-up and storage does not require CAS involvement; these actions are performed using the conveyor belts on FC and Hera & Horstmann and the piston that pushes the part up in WH1.

In order to save space, the evolution of continuous states was represented on the same graph, but what is relevant is the evolution of the state P_c_1_ (black line), which corresponds to the time on the X axis, the interval between origin, and intersection of the line with the axis, about 190 s, for CAS speed of 94 mm/s, which matches the time of the monitoring signal corresponding to state P_35_. 

The handling times of the RM Cyton 1500 and the fine positioning based on the mobile visual servoing system were not considered, both when picking up and storing the part.

The modeling and evaluation of the remaining distance from position (O) until the end of a disassembly–recovery cycle was performed with a continuous Petri net (CPN). In the CPN from Figure 11, the places are associated with the successive positions of the CAS: (A), (B), (C), (D), (E), and (F) for a complete disassembly–recovery cycle of the cylinders, cover and body in the WS4, WS3, and WS4, respectively. The continuous variation of the remaining distance is represented graphically as follows: CAS starts from position (O) (P_c_1_ black)—parking position, passes successively through positions (A), (P_c_2_ red)—(B) (P_c_3_ magenta dark)—(C) (P_c_4_ blue)—(B)(P_c_5_ green)—(D) (P_c_6_ magenta)—(E) (P_c_7_ cyan—(D) (P_c_8_ dark blue)—(F) (P_c_9_ orange), and repositioned in parking position (O) (P_c_1_).

### 3.3. Virtual Digital Counterpart Regarding Repair Function

#### 3.3.1. Assumptions

**Assumption** **R1.** *All conditions and parameters of the repairing function are initially known, including task durations.*

**Assumption** **R2.** *The convention assumes that WP2 does not pass the quality test, being subject to repair if it contains cylinders of different materials.*

**Assumption** **R3.** *WP2 that fails the quality test is stored in the right side of WS6, and its repairing processes starts immediately after.*

**Assumption** **R4.** *The repairing operations of WP2 are executed on FC.*

#### 3.3.2. Task Assignment, Planning, and Synchronization

WP2, having cylinders of different materials (Figure 3d), is taken over by the elevator of WP6 and positioned on WS5. It is transported along the Hera & Horstmann ML to the FC (FC2). The ABB IRM disassembles the plastic cylinder (on FC1), letting it slide on the exhaust chute, and replaces it with a metal cylinder taken from the corresponding warehouse of the FC. 

CAS takes over the cylinder, in any position, 1 or 2, transporting it to the appropriate storage warehouse on the Hera & Horstmann ML. WP2, now having both metal cylinders, is a good quality product, and it is transported from FC, along the Hera & Horstmann ML, to the WS6 station and stored on the left side (Figure 5).

**Figure 13:**
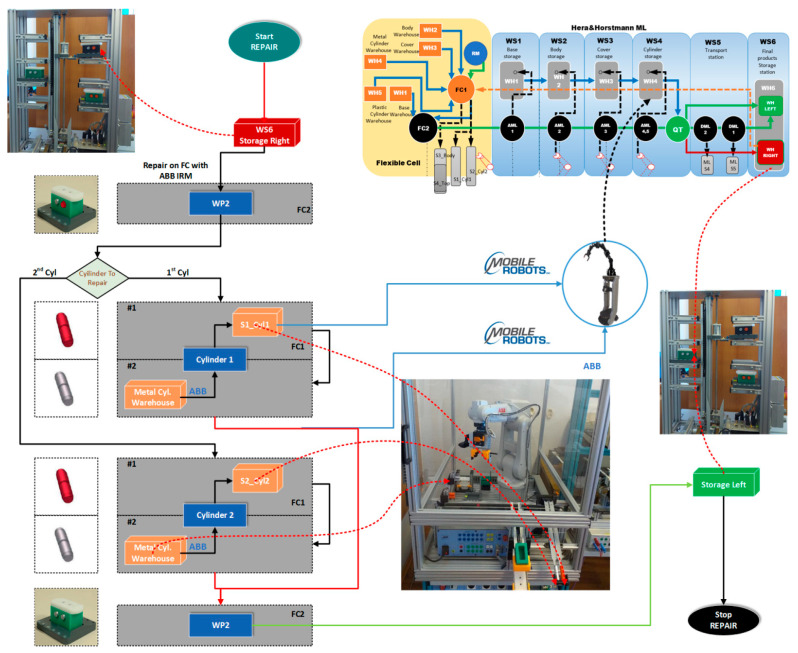
Task planning and synchronization for the repair of the WP2 on FC with ABB IRM.

In Figure 13 is presented the block diagrams with the planning and synchronization of tasks for assembly [28,29,30,31]. The repair functions are associated with the recovery of an assembled final product.

#### 3.3.3. SHPN_2 Model, Formalism, and Simulation

**Figure 14:**
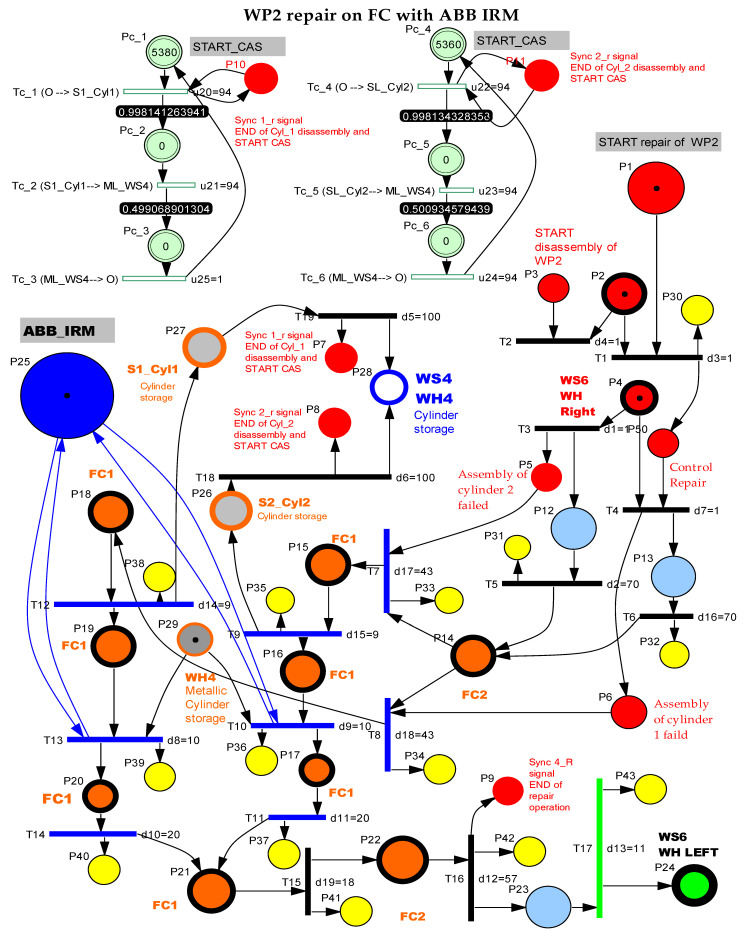
The SHPN_2 model for the repair of the WP2 on FC with ABB IRM.

The SHPN_2 model from Figure 14, based on task planning shown in Figure 13, is an oriented graph described with the synchronized hybrid Petri nets (SHPN) formalism [2,3,12,13,14]. 

The SHPN_2 model describes both discrete and continuous dynamics corresponding to the repair function. The discrete model corresponds to the replacement of the plastic cylinder with a metal one in FC with ABB IRM, while the continuous model corresponds to the CAS displacement for picking up and storing the replaced cylinder in WS4. Thus, the model becomes a hybrid one.

The CAS displacement sequences are synchronized through the synchronization signals (Sync_1_d signal END of Cylinder1 Disassembly and START CAS, Sync_2_d signal END of Cylinder2 Disassembly and START CAS, Sync_3_d signal END of Top Disassembly START CAS, Sync_4_d signal END of Body Disassembly START CAS, Sync_5_d signal END Total Recovery).

The SHPN_2 model, for the disassembly process, is a triplet

(37) $$SHPN\_2=\langle \begin{array}{ccc} HPN, & E^{0^{\prime }}, & Sync \end{array}\rangle ,$$

where: HPN is the hybrid Petri net model, E^0′^ is a set of external events, and Sync is an application.

The HPN is a septuplet:

(38) $$\mathrm{HPN}=\langle T,P,\mathrm{Pr}e,Post,m_{0},h,tempo\rangle ,$$

where

- T is the transitions set partitioned in:

(39) $$T=\left\{T_{d},T_{c}\right\},$$

with

(40) $$T_{d}=\left\{T_{repair},T_{QT}\right\}=\left\{T_{1},..,T_{19}\right\},$$

(41) $$T_{repair}={\left\{T_{i}\right\}}_{i=\bar{1,19}}\backslash \left\{T_{17}\right\}$$

is the set of the discrete transitions for the WP2 repair tasks,

(42) $$T_{QT}=\left\{T_{17}\right\}$$

is the set of the discrete transitions for the quality test (QT) tasks,

(43) $$T_{c}=\left\{T_{c\_1},..,T_{c\_6}\right\}$$

is the set of continuous transitions of the mobile robot (CAS) used to model the continuous repair tasks,

- P is the places set partitioned in:

(44) $$P=\left\{P_{d},P_{c}\right\},$$

where

(45) $$P_{d}=\left\{P_{ctr},P_{repair},P_{monitoring}\right\}={\left\{P_{i}\right\}}_{i=\bar{1,43}}$$

with

(46) $$P_{\mathrm{ctr}}={\left\{P_{i}\right\}}_{i=\bar{1,11}}$$

representing the set of places associated with the control functions related to some decision-making actions,

(47) $$P_{\mathrm{repair}}={\left\{P_{j}\right\}}_{j=\bar{13,29}}$$

the discrete places set modeling the repair operations on the flexible cell (FC),

(48) $$P_{\mathrm{monitoring}}=\left\{P_{30},\ldots ,P_{43}\right\}$$

represents the set of places associated with the monitoring function of the successive repair actions,

(49) $$P_{c}=\left\{P_{c\_1},..,P_{c\_6}\right\}$$

represents the set of continuous places of the mobile robot in repair actions.

Each place in (48) monitors a certain transition in the set (41) as follows: P30(T1_monitoring), P31 (T5_monitoring), P32 (T6_monitoring), P33 (T7_monitoring), P34 (T8_monitoring), P35(T9_monitoring), P36(T10_monitoring), P37(T11_monitoring), P38(T12_monitoring), P39(T13_monitoring), P40(T14_monitoring), P41(T15_monitoring), P42(T15_monitoring), P43(T17_monitoring).

- $Pre:P\times T\to Q_{+}$ is the input incidence function.
- $Post:P\times T\to Q_{+}$ is the output incidence function.
- $tempo:T\to Q_{+}\cup \left\{0\right\}$ is function that defines the time durations associated to the transitions.
- $E^{0^{\prime }}$ is a set of external events, where:

(50) $$E^{0^{\prime }}⊇\left\{Sync1\_r,Sync2\_r,Sync4\_R\right\},$$

(51) $$E^{0^{\prime }}={\left\{E^{i}\right\}}_{i=\bar{1,3}}\cup \left\{e\right\},$$

(52) $${\left\{E^{i}\right\}}_{i=\bar{1,3}}={\left\{Sync\;i\right\}}_{i=\bar{1,3}},$$

(53) $$Sync\;1\_r:T_{19}\to \left\{Ed^{1}\right\},$$

(54) $$Sync\;2\_r:T_{18}\to \left\{Ed^{2}\right\},$$

(55) $$Sync\;3\_R:T_{16}\to \left\{Ed^{3}\right\}$$

{e}, represents the neutral event that is considered to “synchronize” transitions $T\backslash \left\{T_{18},T_{19},T_{10},T_{16}\right\}$_,_

$Ed^{1}\equiv Sync1\_A$ synchronization signal for: (END of Cyl_1 disassembly) with (START CAS),

$Ed^{2}\equiv Sync2\_A$ synchronization signal for: (END of Cyl_2 disassembly) with (START CAS), 

$Ed^{3}\equiv Ed^{4}\equiv \mathrm{Sync}\;4\_R$ synchronization signal for: (START assembly of WP1) with (END repair of WP2) the synchronization signal in the SPN model of the assembly processes.

The repair process is conditioned by the WP2 quality test. If it results that in WP2, the assembled cylinders are different, plastic and metal, $P_{2}$ is directed to the *Repair* sequence. In the process model the following sequences are considered:

WP2, after the quality test in the 4th station of the ML, is deposited in the warehouse WH Right ($P_{4}$ in Figure 14).

- The QT1 and QT2 sequences check the registry of the quality test. Thus, QT1 and QT2 activate one of the $P_{2}$ and $P_{3}$ controls that launches the corresponding repair sequence.
- WP2 is transported to the FC1 station of the flexible cell (P15, P18) where the plastic cylinder is replaced with the metal cylinder. In the S1 or S2 trough, the extracted cylinder is released and is then transported by CAS to the WS4 storage on the ML. WP2, thus repaired, will follow the storage sequence in WH Left.
- The presence of the plastic cylinder in S1_Cyl2 or S2_Cyl2 is synchronized with CAS travel for Cylinder 1 recovering or CAS travel for Cylinder 2 recovering, by {Sync1_r,Sync2_r}.
- The completion of the repair process is synchronized with START a new assembly, through the signal $E^{4}\equiv Sync\;4\_R$, which is found in the SHPN model of the assembly.

**Figure 15:**
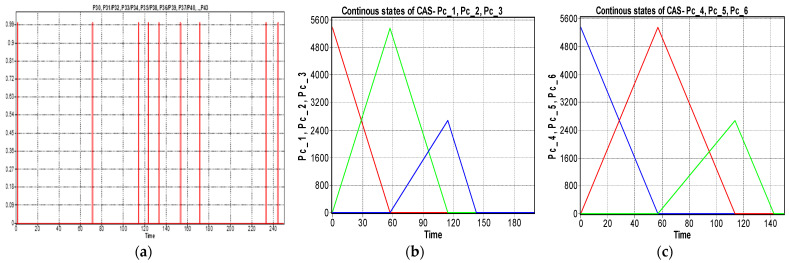
(**a**) The monitoring signals for the successive actions of the WP2 repair. (**b**) The continuous places evolution of the CAS (PeopleBot WMR), places: $P_{c\_1},P_{c\_2},P_{c\_3}$ for replacing cylinder 1. (**c**) The continuous places evolution of the CAS, places: $P_{c\_4},P_{c\_5},P_{c\_6}$ for replacing cylinder 2.

The repair monitoring signals are represented in Figure 15a and the continuous places evolution of the CAS for replacing cylinder 1 in Figure 15b or cylinder 2 in Figure 15c; all of them are obtained via simulation in Sirphyco [32].

In Figure 14, with continuous PN (CPN), is modeled the distance traveled by CAS corresponding to the repair function, which consists of replacing the plastic cylinder in position 1 or 2, taking it from S1 or S2, and storing in WS4. The distance traveled by CAS for cylinder 2 recovery is slightly smaller. CAS takes over from S1 or S2, cylinder 1 or cylinder 2 component, moves to WS4, deposits the cylinder in the WS4, returns, and repositions in the parking position, point (O).

In the two models with CPN, the remaining distance for CAS was considered to have a continuous variation over time. CAS performs the following constant speed travel sequences:

- moving from the parking position $\left(P_{c\_1}\equiv (O)\right)$ or $\left(P_{c\_4}\equiv (O)\right)$ to the drawer S1 $\left(P_{c\_2}\equiv (A)\right)$ or S2 $\left(P_{c\_5}\equiv (C)\right)$, where OA is in red and OC in the blue line, Figure 15a,b, respectively;
- moving from $\left(P_{c\_2}\equiv (A)\right)$ or C $\left(P_{c\_5}\equiv (C)\right)$ to WS4 $\left(P_{c\_3}\equiv (B)\right)$ or $\left(P_{c\_6}\equiv (B)\right)$, where AB is in green and CB in the blue line, Figure 15a,b, respectively;
- moving from WS4 $\left(P_{c\_3}\equiv (B)\right)$ to the parking position $\left(P_{c\_1}\equiv (O)\right)$ or from WS4 $\left(P_{c\_6}\equiv (B)\right)$ to the parking position $\left(P_{c\_4}\equiv (O)\right)$, where BO is in blue and in the green line, Figure 15a,b.

### 3.4. Virtual Digital Counterpart Regarding CAS

This approach will use the mobile part of the A/D/RML, called CAS with Mobile Robots’ PeopleBot WMR, which has an odometer system, two drive wheels, and a rear freewheel. In addition, an embedded microcontroller on board is able to read position information and send it, via the WI–FI link, to a remote PC, according to a specific protocol. The SCADA application from the remote PC calculates the control input and sends it to the WMR. The remote PC also sends the data to the A/D/RML PLCs [2,3]. 

**Figure 16:**
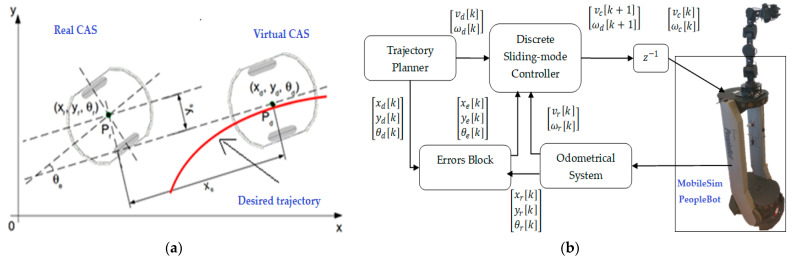
(**a**)-Real and virtual CAS along the desired trajectory, (**b**)-CAS closed loop control in MobileSim or physically.

To control the CAS and the movement between parking, grasping, and placing positions, dedicated functions from the Advanced Robotic Interface for Applications (ARIA) programming package are used and the trajectory tracking sliding mode control (TTSMC) method is implemented, Figure 16a,b. 

**Figure 17:**
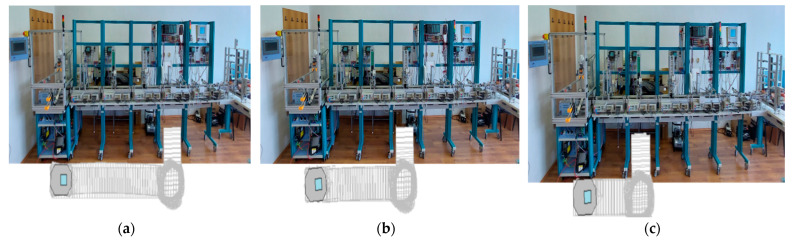
Forward and backward CAS’s trajectories to transport disassembled components to appropriate warehouses: (**a**) plastic cylinders, (**b**) cover, (**c**) body.

Using the MobileSim software described in [8,33], it was possible to test the closed loop control of mobile robots in trajectory tracking by simulation [28,29,30,31]. If no mobile robot is detected, the Aria software will automatically connect to the MobileSim simulator. Figure 17 shows the CAS forward and backward closed-loop trajectories, obtained in MobileSim, for the transport of disassembled components to the appropriate warehouses corresponding to the disassembly function.

## 4. Real Counterpart Control of Multifunctional Technology Running on A/D/RML Assisted by CAS

### 4.1. Real Counterpart Communication and Control of A/D/RML

**Figure 18:**
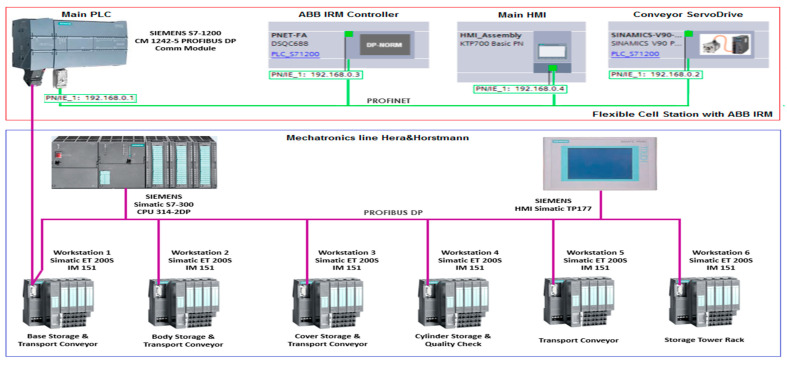
Real counterpart of A/D/RML communication and control architecture.

The real-time control structure of A/D/RML assisted by CAS is shown in Figure 1. The use of the “Supervisory Control And Data Acquisition (SCADA)” system, together with the functionalities implemented in HMI and Remote PC, allow viewing, monitoring, and control of A/D/RML assisted by CAS, through the following actions [3]:

- data acquisition through digital and analogue I/O from FC, A/DML, and CAS for monitoring and control of all sensors;
- data communication, to and from FC, A/DML, and CAS, in which sensors, PLCs, and Remote PC are involved, is necessary for monitoring and remote control of multifunctional technology from a single location through the network of communications. Siemens CM 1242-5 attached to PLC S7-1200 (see Figure 1 and Figure 18) is a device used for communication, having the role of connecting the master PLC (SIEMENS PLC S7-1200), located in FC, with A/DML through Profibus. The SIMATIC S7-1200 module allows connection as a slave to Profibus DP through the CM 1242-5 device that complies with IEC 61158 standards. Thus, it manages traffic autonomously and relieves the main PLC, PLC S7-1200, of communication tasks. CM 1242-5A manages communication data in two directions, one physical and another data link, processing the s signal it receives or sends, and validates the cyclic data transfer between DP master S7-300 PLC from A/DML slave Profibus DP for process data transfer between Profibus DP slaves [3,34];
- displays data and information on SIEMENS HMI SIMATIC KTP 700, HMI TP 177, and Remote PC is performed in a format readable by the human operator through friendly and suggestive GUIs that facilitate a more efficient interaction between the operator and related subsystems: FC, A/DML, and CAS. This represents one of the needs and attributes of Industry 5.0 (see Figure 1);
- remote control, in the network, through SCADA Remote PC, of the devices in the distributed system, of the pending outputs and the synchronization signals from the PLCs, thus facilitating the quick intervention of the operator.

The purpose of the network architecture for the interconnection of the FC, A/DML, and CAS subsystems, presented in Figure 1 and Figure 18, is to provide a unified architecture (UA) and an open communication platform (OPC). OPC UA is a communication data structure between the PLC SIEMENS S7-1200, HMI KPT 700, and SCADA Remote PC that allows integration in other laboratory or industrial systems, which have specific communication protocols. OPC UA also allows connecting to the cloud and using IoT. A/D/RML assisted by CAS also runs with several communication protocols: Profibus, Profinet, Modbus, and Ethernet/IP.

The SHPN models corresponding to assembly, disassembly, and repair are transposed through the Siemens SCADA platform into a real-time application, obtained by interfacing them with synchronized signals taken from the real process through PLCs and sensors [35,36,37]. 

**Figure 19:**
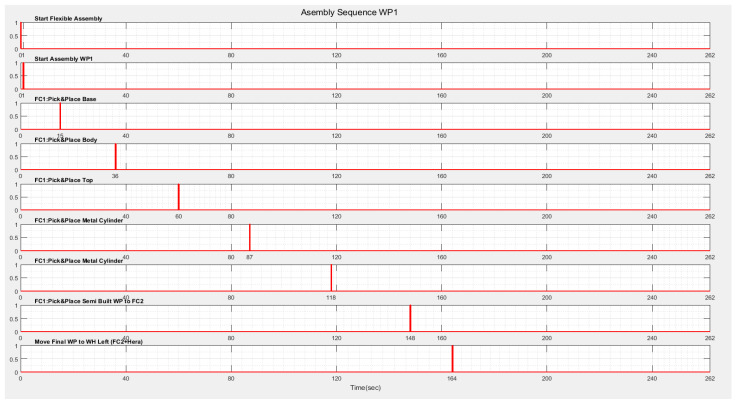
Real-time monitoring signals corresponding to assembly operations of WP1.

**Figure 20:**
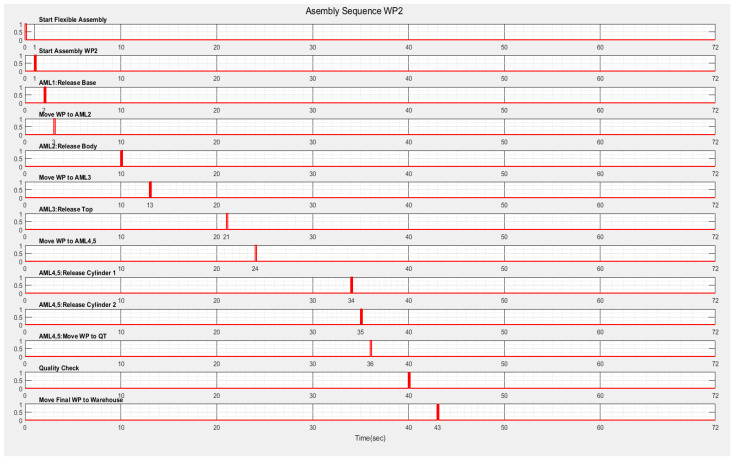
Real-time monitoring signals corresponding to assembly operations of WP2.

**Figure 21:**
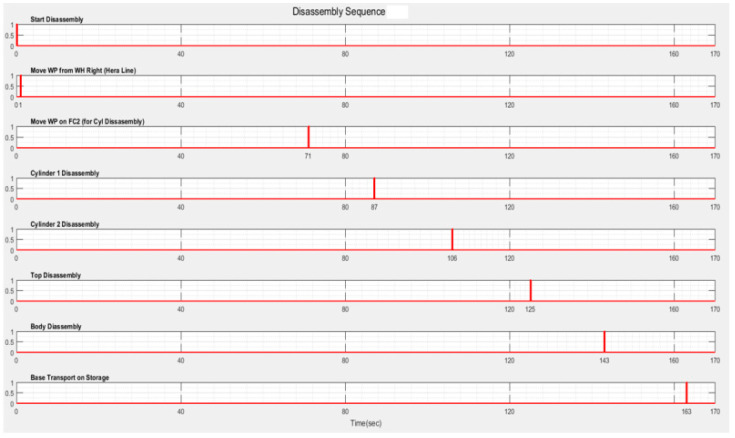
Real-time monitoring signals corresponding to disassembly operations of WP2.

**Figure 22:**
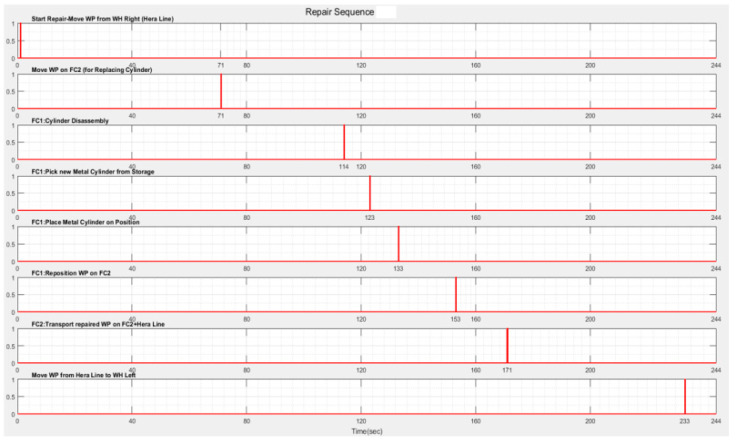
Real-time monitoring signals corresponding to repair operations of WP2.

After implementation, some results of the real-time control A/D/R/ML assisted by CAS are shown in Figure 19, Figure 20, Figure 21 and Figure 22 and are compared with the monitoring signals obtained by simulation, in Sirphyco, of the continuous and discrete states of the corresponding SHPN models, as shown in Figure 9, Figure 12 and Figure 15.

The timing signals, used in the real-time control application, validate certain transitions in the SHPN models [12,13,14]. These transitions are conditioned by the associated signals for the release and recovery of the disassembled components in the FC and their storage in the A/DML warehouses by the CAS. 

Synchronization will result in initialization of the robot and monitoring and control of assembly, disassembly, and repair operations with CAS. Discrete time TTSMC method, based on the kinematic model, is used to control the PeopleBot WMR. In this way, both CAS and A/D/RML are controlled to achieve a minimum cycle time of assembly, disassembly, and repair.

### 4.2. Real Counterpart Syncronisation and Control of CAS’s Subsystems

As mentioned in the introduction, CAS can be assimilated to an MCPS that includes several hardware and software subsystems, deeply intertwined, and able to operate on different spatial and temporal scales. 

The subsystems of CAS are: the PeopleBot as mobile part (2DW/1FW) WMR, the manipulator, the 7-DOF Cyton 1500 RM, and the mobile visual servoing system, referred to as eye in hand VSS, having as a visual sensor a Logitech high-definition (HD) camera. Because in embedded systems the emphasis tends to be more on the computational elements, and less on an intense link between the computational and physical elements, CAS seems to be similar to the Internet of Things (IoT), sharing the same basic architecture, and presenting a combination and coordination between physical and computational elements. The PeopleBot WMR has a built-in microcontroller on board that is capable of reading position information and sending it via the Wi–Fi link to a remote PC, according to a specific protocol. 

The SCADA application from the remote PC calculates the command and sends it to the WMR for traversing the remote PC, then sends the data to the A/D/RML PLCs. To control CAS and WMR movements between park, grab, and place positions, dedicated functions from the ARIA programming package [30] are used, and the TTSMC method is implemented [14,15,29,30]. Based on the IKC, the remote PC calculates the order for the Cyton 1500 RM for parking and positioning related to pick-up and rough plating operations. Based on the method of moments of the image, the remote PC calculates the command for the fine positioning of the end effector on the Cyton RM for picking up and storing disassembled components.

CAS control is based on three control loops, each of which communicates through remote for controlling PeopleBot WMR, Cyton 1500 RM, and MVSS. Remote PC function is like a SCADA server and manages the synchronization and coordination between FC, A/DML, and CAS.

- PeopleBot WMR travel control loop is for moving from FC to storage warehouses of disassembled components and from FC to their placement on dedicated storage warehouses on A/DML. The control method is discrete-time TTSMC. The functions from Aria Mobile Robots are called. Communication with the FC is performed wirelessly using a USB over Ethernet 704 adapter and a specific TCP/IP protocol.
- Control loop is for synchronization commands between Siemens S7-1200 1200 and Cyton RM via Modbus TCP signals [2,3]. Communication between the Cyton RM and the Remote PC is performed wirelessly, using Ethernet adapter and a specific TCP/IP protocol [2,15,37,38].
- Control loop for eye-in-hand VSS is based on the image moments method, for end effector movement of Cyton RM. Communication is also performed wirelessly between Remote PC and MVSS, for accurate pick-and-place positioning of the robot [3,39,40,41,42,43,44,45]. The control method calls functions from the OpenCV open-source library [46] and MATLAB image processing toolbox [2,47]. All three control loops communicate through Remote PC, which also acts as a SCADA server, controls the CAS, MVSS, and Cyton RM, and manages the synchronization with the FC and A/DML.

### 4.3. Real Counterpart Control of the MVSS and Cyton RM

The structure of the mobile visual servoing system (MVSS or eye in hand VSS) includes the visual sensor (Logitech HD video camera) and a control loop that works based on the image moments method. The video camera is located on the end effector of the Cyton 1500 (see Figure 1). 

The control loop needs deductive information and controls the system environment to minimize the error between the actual configuration of visual features, f, and a desired configuration, f^*^. In control, MVSS minimizes the error between the real and desired features extracted by the visual sensor [15,16,39,40,41,42,43,44,45,46]. 

**Figure 23:**
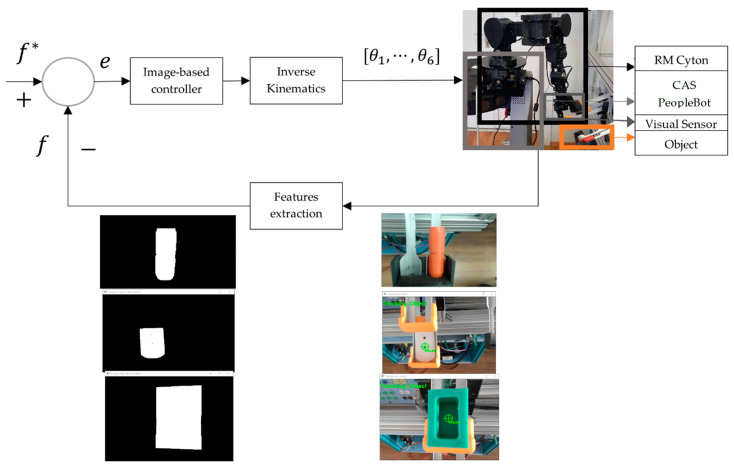
Closed-loop control of the RM Cyton based on eye-in-hand type VSS.

The VSS control loop is shown in Figure 23. The main steps involved in object detection and tracking are shown in [2]. The Cyton 1500 RM with end effector offers robust, intelligent, and precise handling. 

For testing and simulating the Cyton RM, kinematic modeling needs to be performed, having as the main objective the study of RM’s mechanical part regarding the direct and the inverse kinematics. The direct kinematics consist of finding the position of the end-effector by knowing the movements of the articulations,

(56) $$F\left(\theta_{1},\theta_{2},\ldots ,\theta_{n}\right)=\left[x,y,z,R\right]$$

and inverse kinematics consist in determining the value of every articulation by knowing the position of the end-effector and its orientation,

(57) $$F\left(x,y,z,R\right)=\left[\theta_{1},\theta_{2},\ldots ,\theta_{n}\right]$$

An RM is composed, in general, from three articulations, each defined by one or more degrees. In the case of RM Cyton, it is made of:

- one shoulder type articulation, characterized by three angles;
- one elbow type articulation, characterized by one angle;
- one wrist type articulation, characterized by three angles.

### 4.4. Real Counterpart Control of CAS PeopleBot Assisting A/D/RML during Disassembly

The SHPN model in Figure 11, a model that corresponds to the disassembly function, is transposed through the SCADA platform from Siemens into a real-time application, obtained by interfacing the SHPN model with the synchronization signals taken from the real process through PLCs and sensors [2,3]. 

The synchronization signals, used in the real-time control application, validate certain transitions between the states of the SHPN model [3]. These transitions are conditioned by the associated signals for the release of disassembled components on the inclined troughs of FC, recovered, seized by CAS, transported, and stored at the corresponding warehouses of A/DML. Synchronization will result in CAS initialization, monitoring, and control of disassembly operations. Thus, both CAS and A/D/RML are controlled to achieve a minimum cycle time for disassembly. 

To capture the components resulting from disassembly and store them in the appropriate warehouses of A/DML, the gripper is positioned by the VSS so that it grabs the disassembled component, transports, and places it in the warehouse. 

**Figure 24:**
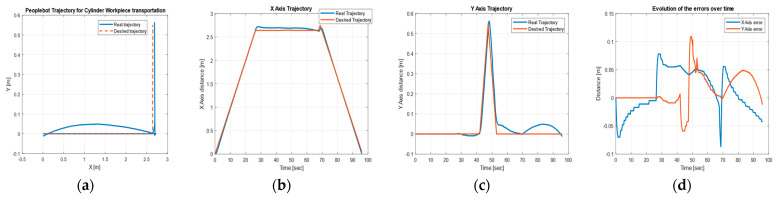
CAS’s DT-TTSMC for cylinder recovery: (**a**) desired and real trajectory; (**b**) along the *X* axis; (**c**) along *Y* axis; (**d**) errors.

Figure 24 shows the desired and real trajectories of CAS obtained with the discrete-time TTSMC method for the movement from FC (S1 and S2, Figure 4) to the warehouse on WS4 on A/DML, along with the trajectories and errors on the X, Y axes, corresponding to the recovery of the cylinders. 

**Figure 25:**
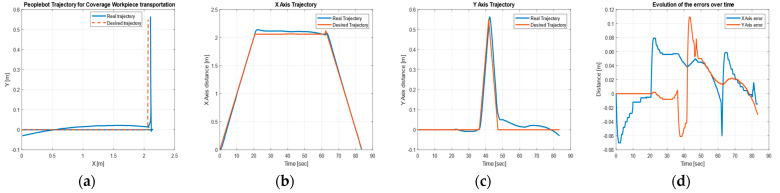
CAS’s DT-TTSMC for top recovery: (**a**) desired and real trajectory; (**b**) along the *X* axis; (**c**) along *Y* axis; (**d**) errors.

Figure 25 shows the desired and actual trajectories of the CAS obtained with the discrete-time TTSMC method during closed-loop driving for the movement from FC (S4) to the warehouse on WS3 on A/DML, together with the trajectories and errors on the X, Y, axes, corresponding to the recovery of the top. 

**Figure 26:**
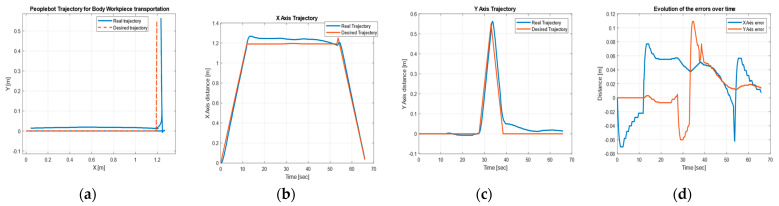
CAS’s DT-TTSMC for body recovery: (**a**) desired and real trajectory; (**b**) along the *X* axis; (**c**) along *Y* axis; (**d**) errors.

Figure 26 shows the desired and actual trajectories of the CAS obtained with the discrete-time TTSMC method during closed-loop driving for the movement from FC (S3) to the warehouse on WS2 on A/DML, together with the trajectories and errors on the X, Y, axes, corresponding to the recovery of the body.

In the “Digital Twin” approach from this paper, for both the virtual and the real world, the following software products and packages were used for simulation, monitoring, and control: Sirphyco [32], Microsoft visual studio C++ [48]; Advanced Robotic Interface for Applications (ARIA) [49]; MobileSim [33], Siemens Totally Integration Automation (TIA-Portal V.15) [34]; Open CV [45]; MATLAB [46].

## 5. Discussions

The monitoring signals of the successive actions for each functionality were: assembly (Figure 9), disassembly (Figure 12), and repair (Figure 15), resulting from the Sirphyco simulation of SPN and SHPN, and are approximately temporally close to the monitoring signals provided by PLC, following the execution of programs in real-time control: Figure 19 and Figure 20 for assembly, Figure 21 for disassembly, and Figure 22 for repair. Thus, the virtual digital component of the multifunctional flexible manufacturing technology is validated in real time. 

The simulation of the continuous states from the SHPN models related to the disassembly and repair is useful in comparing the evolution of the discrete states of A/D/RML with the physical constraints of the CAS in the displacements made for the recovery and storage of the components. A/D/RML, even if it includes an industrial robotic manipulator (ABB 120 IRM) that can handle loads in the order of kilograms, is a laboratory system, and therefore, the components resulting from disassembly or repair, have masses in the order of tens of grams. Thus, the direct and inverse kinematic model basic control of the Cyton 1500 manipulator, for the handling and positioning operations, is robust to uncertainties. 

For precision positioning, when gripping or releasing the part, control of the Cyton manipulator, based on the mobile visual system and moments of the image, proves to be a good choice, being less sensitive to light disturbances.

The following concepts used in this work satisfy the requirements of Industry 4.0 and challenge Industry 5.0 [23,24,25,26,27]:

- Digital twin with augmented reality (AR) component represents the integration of the virtual and real environments, where objects in the real world are enhanced by computer-generated information or objects that help the multi-functional flexible assembly technology. Thus, SHPN was used for technology modeling in which A/D/RML has a discrete dynamic and MCPS a continuous one;
- The “Digital twin” concept enables optimizing the production line and predictive maintenance that can improve efficiency and detect problems in time. By means of the virtual world model as counterpart of the real one, defects and problems that may appear in the production process can be detected in advance;
- The simulation of SHPN and MCPS models is a powerful tool used for decision-making. By using the simulation results, the monitoring and control methods become easier to apply, together with the developments in the field of digitalization;
- The use of MCPS as an autonomous robotic system, equipped with RM and positioning and navigation sensors, represents a complex, next-generation system with computational and physical capabilities that can interact with humans in new ways;
- Artificial intelligence supports MCPS, A/D/RML PLCs, HMIs, and SCADA by filtering sensor data from the manufacturing system, thus providing data-driven predictive analysis and the ability to assist in decision-making;
- Through OPC UA, access to cloud computing and IoT is facilitated, allowing access to large data sets and their processing to generate new useful information for the manufacturing process and multifunctional technology. As I mentioned above, OPC UA is the communication data structure between SCADA and SIEMENS PLC S7-1200, which, integrated in an industrial manufacturing technology, ensures compatibility and safe data exchange between the industrial equipment of the different providers of software;
- Because the multifunctional technology through disassembly and repair functions allows the recovery and reuse of components, it ensures the sustainability of the production system. Sustainability is an important component of Industry 5.0, which focuses on the reuse and recycling of natural resources and reducing waste and environmental impact;
- Since the hardware configuration and management methods of A/D/RML and MCPS ensure the robustness of all subsystems to defects and uncertainties, the resilience of the production system is conferred, being another concept that is specific to Industry 5.0. Robustness provides support through flexible processes and adaptable manufacturing capabilities, especially when a crisis occurs;
- Last but not least, through the graphic user interfaces on HMIs and remote PC, an approach to multifunctional technology, centered on the human operator, was tried, a concept by which Industry 5.0 places human needs at the center of the process, asking what technology can do better and how it can be useful.

## 6. Conclusions

The “Digital Twin’’ approach to multifunctional flexible manufacturing technology is based on several motivations. First, it is due to the existence of several software tools for modeling and simulating all the functionalities of the system. The second is the need for compatibility, both in terms of communications and dynamics of the two major subsystems, A/D/RML and CAS. The third is the ability to remotely monitor the entire process via a graphic user interface. The fourth is the validation of the functionality of the virtual component through real-time implementation and the elimination of discrepancies through direct intervention at the hardware level. 

Thus, the contributions and results obtained would be the following:

- hardware setup, assumptions, flexibility and multifunctionality;
- virtual digital counterpart for each functionality: assumptions, task planning and synchronization, SHPN model and formalism, and simulation results;
- virtual digital counterpart of the CAS: model, control, and simulation results for each component;
- real counterpart control of multifunctional technology: SCADA system, communication and control of A/D/RML, synchronization and control of CAS’s subsystems, real-time results.

We wanted to build a holistic view of all component subsystems by using standards of communication, data acquisition, and control. The system is still open, interoperable, and connectable to IoT and the cloud. Finally, the “Digital Twin” approach has been designed to meet all the requirements and attributes of Industry 4.0 and beyond, toward Industry 5.0. 

This flexible and multifunctional manufacturing technology, assisted by robotic systems and visual servoing able to recover and reuse components, meets the requirements of the real industrial world, to manufacture products with clean technologies and recyclable materials, but with a high degree of precision and quality. 

The control structure is hierarchical and multilevel, with a supervisor at operation level that monitors the process, execution, and synchronization of tasks according to the strategy, followed by, communication level; PLCs level; A/DRML; and CAS control level.

The challenges for new technologies are those that satisfy the requirements of Industry 5.0, technologies that take on a human touch and highlight several concepts, such as cyber resilience, sustainability, environment, purpose, values, ethics, diversity, and a circular economy. The targets of Industry 5.0 are the place of people in a future of work with more human–machine collaboration, human-centric solutions, and, well, also some technical issues. Industry 5.0 recognizes the power of industry to achieve societal goals beyond jobs and growth to become a resilient provider of prosperity, by making production respect the boundaries of our planet and placing the wellbeing of the industry worker at the center of the production process. 

The paper also has a double aspect, educational and research, addressing undergraduate, master’s and doctoral students in control systems, aiming to familiarize them with everything that define a new industry architecture, including Industry 4.0 and 5.0 concepts, and tries to improve the design of current technologies with the integration of all new, state-of-the-art aspects in manufacturing and engineering, including smart and intelligent manufacturing products.

## Data Availability

Data availability is not applicable to this article as the study did not report any data.
